# Between art and information: communicating world health, 1948–70

**DOI:** 10.1017/S1740022817000304

**Published:** 2018-03

**Authors:** Alexander Medcalf

**Affiliations:** Centre for Global Health Histories, Berrick Saul Building BS/120, University of York, Heslington, York, YO10 5DD E-mail: alexander.medcalf@york.ac.uk

**Keywords:** communications, mass media, photography, public information, world health

## Abstract

With the advent of new media technologies and approaches in the twentieth century, public health officials became convinced that health needed mass media support. The World Health Organization believed that educating people, as well as informing them about the health situation around the world, could assist in the enduring fight against disease. Yet in an increasingly competitive media landscape, the agency recognized the need to persuade people and hold their attention through attractive presentation. Public information, the name given to the multiple strategies used to communicate with the public, was rarely straightforward and required the agency not only to monitor the impact of its own efforts but also to identify opportunities to further enhance its reputation, especially when this was in danger of damage or misappropriation. The WHO’s understanding of public information provides insights into the development of international information, communication, and education networks and practices after 1945, as well as the increasingly central position of these processes in generating support for and evincing the value of international organizations.

In the summer of 1956 the World Health Organization (WHO) considered an intriguing proposal from George Englund, a producer and partner in a small Hollywood film company called Pennebaker Productions. Englund sought to produce a feature film for a popular audience based on the technical assistance activities of the United Nations (UN) and its specialized agencies. His interest in international technical assistance had been stimulated some two years previously, when he witnessed the work of the WHO and the UN in South Asia. Afterwards he met with WHO officials to make his case, and by 1956 his team was eager to take the project to the next stage. On the face of it this was not an unusual request for support. By that time the WHO had worked with numerous film and television companies, photographers, and radio producers to document the organization’s activities. But what marked this proposal out was the other name in the picture. Pennebaker was a small studio which traded on the reputation of its only star and part-owner, Marlon Brando, the leading man of box office successes such as *The Wild One* (1953) and *On The Waterfront* (1954). Brando, too, was absorbed by the work of the UN, and saw it as the perfect theme for a high drama extravaganza, with South Asia providing the exotic backdrop.^1^

Officials throughout the different sections of the WHO and the UN were both enticed by and apprehensive about the idea. It was felt that Pennebaker’s pitch was persuasive enough: Brando, Englund, and their writer, Stewart Stern, had all impressed as individuals who were intelligently and earnestly interested in developing the film. Prior to Brando’s scheduled film work in Japan, the group had reconnoitred a number of countries where technical assistance programmes were underway. Furthermore, it was noted that there was little preventing Pennebaker or indeed anyone else from making a film inspired by the UN. However, official backing was a different matter. From here the project began to falter, largely because of the fundamental need to protect the identity and integrity of UN agencies. While a high-profile film might have served the WHO well in making its mission and work known on a grander scale and to new audiences, it also had the potential to harm the agency’s reputation. The Pennebaker group was advised that ‘undue and highly-coloured’ publicity of the WHO’s activities in a country would be undesirable, and that they should be sensitive to the cautious attitude which UN officers had to present in the field. The WHO’s director general, Dr Marcolino Candau, warned that ‘any misconceptions which might be fostered by such a film would be harmful, whether a particular agency was mentioned or not’.^2^ Ultimately the project went no further within the UN, and in July 1959 Brando was still searching for the right script.^3^ Pennebaker eventually released a version of the film in 1963 as *The Ugly American*, based on the bestselling 1958 novel by Eugene Burdick and William Lederer. In this version, Brando played Harrison MacWhite, the United States ambassador to Sarkan, a fictional Southeast Asian nation on the brink of civil war.^4^

The coming together of the WHO and Marlon Brando, albeit a minor footnote in the history of the silver screen, has wider significance with regard to the attempts made by international organizations to communicate their existence and represent their work to different audiences around the world. ‘Public information’ was the term given to the different materials and strategies, including popular films, that were used by such organizations to raise awareness and foster understanding of their role, as well as to mould public sentiment. This could not be achieved simply by producing and disseminating information; publicity officers were often required to creatively frame ‘information’ in exciting, touching, and memorable ways. This article examines the public information machine, exploring what the WHO understood by public information and what it intended it to do; how campaigns were structured and managed in light of contemporary tensions about the use of propaganda; and how these strategies were evaluated. It will discuss how the agency managed its image, as well as how it safeguarded its brand and reputation. The WHO example is illustrative of the complex process of representation in which international organizations were engaged. These campaigns required judicious use of information and persuasion to target audiences who did not consume material uncritically and were increasingly suspicious of the motives behind official communications.

The article finds that the WHO’s public information work had different but related purposes. The first was to assist in developing an informed public opinion among all peoples on matters of health, on the basis that the public could not be expected to give support without accurate information regarding the problems that the WHO tackled. The second function was to rouse and satisfy a general interest in the WHO and its work, which went beyond disseminating information to showing the agency in a positive light. These two goals overlapped and merged, and together display a different facet to international health in this period, revealing attitudes that wavered between providing health education and prioritizing public relations.

The Second World War compelled governments to organize mass media campaigns to keep citizens informed about and loyal to the war effort. However, the rise of false and manipulative propaganda tarnished what had once been a neutral term and tainted the image of public information. As such, while it became a significant component in the UN’s post-war drive for international peace and cooperation, the specifics of operation and intentions in public information campaigns were obscured. Mark Alleyne’s detailed history of the UN’s public information work throughout the second half of the twentieth century notes a constant tension: although the role of public information was to counter the threat of misinformation and destructive propaganda, the organization was equally concerned that its own efforts would be construed *as* propaganda, thereby damaging its international reputation.^5^

At the same time, many of the campaigns released by the UN’s specialized agencies were designed to persuade and influence rather than soberly inform, epitomized by the growing centrality of visual appeals. Tom Allbeson and Zoë Druick demonstrate how the United Nations Educational Scientific and Cultural Organization (UNESCO) embraced visual media strategies as a way to overcome national boundaries, inculcate mutual understanding, and garner support for the agency’s work.^6^ UNESCO sought to facilitate a ‘right’ way of looking at the world and its problems, and inspire a sense of obligation through, for instance, photographs of children.^7^ The extensive photograph campaigns of the United Nations Children’s Fund (UNICEF) similarly focussed on recognizable and emotional tropes, such as alternately harrowing and uplifting photographs of mothers with children, to construct a visual vocabulary of ‘hunger’ and ‘need’ which was used to generate support and donations for its mission.^8^ UNICEF convened large-scale public relations campaigns in the United States, including ‘trick or treat for UNICEF’, and used celebrities such as Danny Kaye and Peter Ustinov as goodwill ambassadors.^9^ Although it is difficult to judge the precise nature of the impact, these high-profile campaigns probably played a significant role in influencing how people around the world perceived certain issues, countries, and peoples in ways that went beyond an awareness of their plight and needs.

Official histories of the WHO confirm not only the importance of public information but also the rich diversity of materials and strategies used to shape popular attitudes towards illness and disease, which included cartoons, radio series, exhibitions, and world health observation days.^10^ As the most popular leisure pursuit in the immediate post-war years, film was exceptionally important, and Kirsten Ostherr has examined a number of WHO films to determine how they influenced popular understandings about the global spread of disease.^11^ The era also witnessed the unprecedented popularity of weekly photo magazines: Bert Hansen argues that, before the mass availability of television, *Life* magazine was instrumental in creating visual archetypes of nurses and medical researchers, which became so familiar to the public eye that they took on the status of icons.^12^ The WHO published its own illustrated magazines that mirrored the style of *Life*, and the agency’s published and unpublished photography includes a truly vast range of stories, countries, and photographers, including archival files for more than 800 photo missions conducted between 1953 and 1989.

Thomas David and Davide Rodogno have led the way in opening up the extensive WHO photographic legacy to scrutiny. They have revealed information about the nature of collaborations with outside agents such as the celebrated Magnum photo agency, and have identified particular narratives in the agency’s work up to the late 1960s.^13^ They argue that the WHO relied on photographs to construct ‘before and after’ narratives which precisely demonstrated the efficacy of medical interventions and, by extension, the important work of the WHO. Photo stories also drew upon recognizable and understandable themes about development and progression, centred on a cast of villains, victims and saviours.^14^ These narratives enabled the WHO to give its technical assistance work a human face, allowing it to portray incontrovertible proof of its success and thereby enhancing its authority.

While films and photographic campaigns were central pillars of the WHO’s communications, they were part of a larger public information machine set up to inform the ‘greatest number of people within the general public about the Organization’s work and about health matters more generally’.^15^ This article builds on the work of David and Rodogno in a number of key areas. It reconstructs the agency’s attitude to and development of public information campaigns across roughly the first two decades of the WHO’s operation until the 1970s, and shows how this impacted on the design and distribution of the WHO’s magazines. It locates the agency’s photographic output within this public information context to add information regarding how certain visual narratives were built up and propounded. A key strategy in relation to photographs was to distribute them as widely as possible, and the article therefore examines the mechanisms used to do this, the consequent reach of the images, and their afterlife beyond the WHO. Unpublished notes and contact sheets from photographic missions, in conjunction with published variants, demonstrate that, despite its appreciated capacity to provide an accurate pictorial record, photography was used primarily emotionally, in order to sensitize the public to issues that the WHO thought important. Photography therefore represents a key way to a better understanding of how the WHO approached its public information work. However, this was not without its problems: the discussion surrounding the Brando film project is just one high-profile instance that demonstrates the WHO’s early interest in projects which embraced popular visual culture as a means to propagate messages about science, health, and development, but that the organization was also anxious to control the nature of such messages.

## The rise of public information

The beginning of international cooperation in the field of health is commonly traced to the organization of the Sanitary Conferences, the first of which was held in 1851. However, by the late nineteenth century the tide of opinion held that a permanent regulatory body would be better placed to oversee matters in the long term. An International Sanitary Bureau was subsequently formed in 1902, followed by the Office International d’Hygiene Publique in 1907.^16^ After the First World War, the League of Nations Health Organization (LNHO) was given a broad remit to prevent and control the spread of disease, and to examine health problems relating to nutrition, housing, and rural hygiene. The LNHO operated with mixed success throughout the interwar years, and its work was all but halted during the Second World War. In April 1945, the Allied nations assembled at the United Nations Conference on International Organization to debate the maintenance of peace and security through international cooperation on economic, social, cultural, and humanitarian problems. However, these discussions did not at first envisage an agency specializing in health. This omission prompted the Brazilian and Chinese delegations to propose the amalgamation of existing health organizations into one specialized agency, recognizing that health was a fundamental pillar of international peace and security and should be formally acknowledged as such.^17^ With the UN ultimately agreeing upon the value of such an agency, the International Health Conference was convened in New York between June and July 1946 to draft the constitution of the WHO and arrange an interim commission to preside over health matters until the agency was established. The constitution came into force on 7 April 1948. That date would henceforth be known as ‘World Health Day’, to commemorate the agency’s formation and to direct international attention towards urgent health problems. As well as a central headquarters at the Palais de Nations in Geneva, regional offices were established in Africa, the Americas, Southeast Asia, Europe, the Eastern Mediterranean, and the Western Pacific.^18^

The WHO’s role was to provide technical assistance to UN member states, meaning that it would not intervene directly but work with governments to develop efficient health services, monitor outbreaks of disease, promote health education, and coordinate research. Its constitution proclaimed good health to be the right of every human regardless of race, religion, political belief, or economic or social condition, and defined health ambitiously as a state of complete physical, mental, and social wellbeing and not merely the absence of disease or infirmity. However, from the very start opinion was divided over how this could and should be achieved. The WHO’s first director general, Brock Chisholm, together with key figures in the WHO’s formation such as René Sand, Andrija Stampar, and Karl Evang, was a proponent of social medicine, which advocated a slower, holistic approach to health, linked to broader development.^19^ Yet the Second World War had delivered new ‘weapons’ in the fight against disease, in the form of antibiotics, vaccines, and insecticides, which promised quick resolutions.

This tension between targeted, rapid interventions and the more measured and extensive social medicine approach was heightened by Cold War suspicions: while the Soviet Union favoured social medicine, it was distrusted by the United States, which insisted that vertical programmes and rapid-action projects provided the best chance of success. The United States also believed that the impressive new medical weapons could be used to counter the spread of Communism by winning the hearts and minds of countries facing ingrained health problems.^20^ When the Soviet Union and the communist nations in its sphere of influence boycotted the UN system in 1949 (returning in 1956), the United States became freer to influence the WHO’s work priorities.^21^ Disease eradication campaigns against malaria, tuberculosis, and venereal diseases became prominent and well-covered initiatives in WHO’s arsenal.

Although such programmes were prominent, it was felt that the success of the WHO should not depend solely on them, and the agency’s constitution called for the development of informed opinion on matters of health.^22^ The envisioned audience was not just medical and public health professionals but all humankind. Brock Chisholm spoke of his desire to instil a ‘world health consciousness’.^23^ Becoming operational in September 1948, the WHO Public Information Office (PIO) was tasked with helping to achieve this by presenting and interpreting the activities of the agency for the public. Its role was to ensure the readability and uniformity of style of publications; disseminate the findings of technical divisions; satisfy requests for information from publicity media and international, governmental, professional, and civic organizations; and help combat sensationalist reporting and misinformation on health matters.^24^

By this time, public information was of acknowledged importance, explaining why a dedicated office was formed so early in WHO’s history. In the early years of the twentieth century, new psychological theories about how to influence public attitudes had raised the profile of public information. The burgeoning mass media environment afforded greater opportunity to influence public opinion, but it also meant that organizations competed for attention and support in an increasingly crowded marketplace. Francesca Piana shows that, facing competition in the humanitarian realm, the International Committee of the Red Cross (ICRC) established a Propaganda Commission in 1919 to advertise its activities through photographs and film. Such campaigns provided evidence of the ICRC’s work, yet sought to draw audiences to its cause by displaying unequivocally the plight of suffering and rescued peoples.^25^ During the interwar years, the Save the Children fund spent 5% of its annual income on advertising in national broadsheets and radio broadcasts, and published a bi-monthly magazine titled *The world’s children*.^26^ High-ranking figures in the League of Nations stressed that ‘the public all over the world will have to be taught to think internationally, to look at public affairs, not merely from the sectional national point of view, but also from a broad human international point of view’.^27^ Heidi Tworek shows that, for the League, public information strategies represented a key tool to counteract the spread of false information, encourage international cooperation, and assist with the ‘moral disarmament’ of all nations.

However, the WHO PIO was inaugurated at a time of heightened suspicion surrounding public information. As Mark Wollaneger points out, before the Second World War ‘propaganda’ was often used in a neutral sense in relation to advertising, but during the conflict it became linked to more sinister motives of deception and coercion.^28^ Yet, although terms such as ‘information’, ‘propaganda’, and ‘publicity’ continued to be used interchangeably behind closed doors, the war alerted the public to the potential dishonesty of information.^29^ Alleyne’s work demonstrates that, while the UN Department of Public Information (DPI) was formed in 1946 to inform and educate citizens about global problems and priorities, and to sensitize them to the benefits of mutual understanding and collaboration, it was equally concerned about accusations of ‘propaganda’.^30^ Explaining the department’s functions in the *Public Opinion Quarterly*, Benjamin Cohen, the first assistant secretary general in charge of the DPI, noted that it would ‘collect, collate and give out factual information concerning the United Nations on an international basis’, but stated categorically that it was ‘not a propaganda agency’.^31^

In line with the common information policy envisaged by the UN and its specialized agencies,^32^ the WHO similarly demanded that all its information should be ‘presented in an objective way, avoiding any intention of propaganda’.^33^ Yet internally the lines were blurred, since the need to persuade as well as inform was frequently acknowledged. The terminology used to explain the concept was varied: a 1947 report by the WHO’s Interim Commission described public information activities as ‘simply another media of public relations’;^34^ a 1955 public information study stated that, while public information should be used to ‘promote knowledge and understanding of the reasons for the Organization’s existence, aims and its activities’, it needed to combine ‘strict scientific accuracy’ with ‘attractive and popular presentation of material’.^35^ In the official document recording the WHO’s first ten years of operation, the basic function of public information was described as being to make ‘the facts easily available in an appropriate and acceptable form’ because the agency’s policies and programmes ‘will be appreciated only if the scientific facts underlying them are made widely known’.^36^ Yet readership should also be ‘influenced’ by coverage that combined technical accuracy with a presentation sufficiently attractive to engage and hold the interest of the general public.^37^

Early strategies addressed the dilemma of priming the public to be interested in the WHO’s work and amenable to its goals while remaining objective and impartial. ‘Basic’ public information materials included those which were ‘written, oral or visual, explaining the history, purpose, philosophy, structure and methods of work of the Organization, and describing various health activities which are its concern’.^38^ For internal purposes, this documentation was divided into ‘general documentation’ and ‘specialized reporting’, although it was acknowledged that no hard and fast distinction was possible.^39^ The folder titled ‘WHO, what it is, what it does, how it works’ provided essential facts about the WHO’s operation, while booklets such as ‘Strategy for world health’ and the more romantically titled ‘The lamp is lit’ presented the story of the agency and its practical work in a popular form. An illustration of the type of undertaking that resulted from these steps was the externally published 1956 book for young people, *Mankind against the killers*, authored by James Hemming. The outcome of discussions held in 1953,^40^ it contained a foreword by Brock Chisholm and, although it dealt with international health work in general, many sections eulogized the WHO’s existence. Chapter 14, ‘The front line’ began:

On a magnificent site near one end of the Lake of Geneva, and favoured with a glorious view of lofty Mont Blanc on the far side of the lake, stands a gracious and commanding building. It is the property of no nation, no government, no wealthy business syndicate; it belongs instead to everyone – to mankind. … All day long the Palais hums with activity. Its staff, recruited from forty different nations, have a constant full schedule of work. Visitors of all nations and colours come and go; they bring the health problems of their own countries and take away the best advice the world has to offer …^41^

While enhancing the WHO’s public image would not be discussed seriously until the 1970s, the issue was present right from its inception, evinced in the orchestration of public-oriented publications as well as the design and management of an emblem (comprising the symbol of the United Nations surmounted by an Aesculapian staff and serpent in gold), and in the measures taken to safeguard against unauthorized usage of the WHO’s professional identity. Although allusions to propaganda were publicly played down, these efforts served an important public relations role and helped to establish the WHO as an acknowledged authority on health matters.

In the years before the professionalization of dedicated health correspondents, medical advances or disease outbreaks could be reported erroneously in the popular press.^42^ While this was impossible for the WHO to control, the agency sought to balance the flow of information by relaying the organizational viewpoint and making the facts ‘easily available and in an appropriate and acceptable form’.^43^ For health measures to have lasting value, people of all ages needed to be persuaded to take an intelligent and responsible part in solving their personal health problems and those of the community.^44^ The PIO’s activities therefore aimed to downplay scaremongering and miraculous promises, and to nurture public interest in health matters. Officials recognized that this could not be achieved through the limited reach of WHO publications alone, and the stimulation of outside coverage was considered a specialist task which, in view of budgetary restraints, was the only hope of featuring the WHO in the ‘expensive’ media. Yet professional reporters could not be ordered to cover the WHO, and the results of their work could not be guaranteed. Selected writers, journalists, and producers were therefore invited to visit the WHO’s Geneva headquarters, and travel fellowships were arranged but financed externally.^45^ Sensitive to the charge of propaganda, the WHO imposed the condition that the resulting materials should be the work of these independent ‘witnesses’. In 1967 more than 2,000 people visited the WHO’s headquarters in the orderly and impressive Palais des Nations.^46^

To further facilitate this process, the WHO published its own public-oriented magazines, first the *Newsletter* and later *World Health*. The *Newsletter* was inaugurated in September 1948 as a small, typewritten publication of six pages without illustrations. It rapidly underwent stylistic changes and a revamped *Newsletter* was introduced in March 1949, ‘primarily for individuals and institutions, whether public or private, with an interest in the work of the WHO or in international co-operation on health matters generally’.^47^ It was nevertheless welcomed by field staff, who felt isolated from the organization’s wider activities. The *Newsletter* was distributed gratis from the WHO headquarters and regional offices in response to individual requests, and by 1955 60,000 copies were circulated.^48^ Its successor, *World Health*, represented a further step away from simply satisfying the demand for information and towards inciting and shaping interest. Its official purpose was to ‘stimulate and nourish interest in international health matters among the general public’,^49^ and therefore the criteria governing the selection of material were popular appeal and general attractiveness, where the human interest angle was usually ‘played up’.^50^

By 1959, *World Health* had circulated some 80,000 copies in English, Spanish, French, and Portuguese; a decade later this had increased to around 120,000.^51^ Arrangements were also made for issues to be translated into Russian, and the German Green Cross published a German edition. Appreciating that there was a realistic limit to the reach of its magazines, the WHO hoped that the content within would catch the eye of mainstream media editors and journalists. To expedite this process, articles and photographs printed in both magazines were made freely available for reproduction: ‘Are you an editor? If so has it ever struck you that some of the stories you read in this *Newsletter* would also interest the readers of your newspaper or periodical? You are at liberty to reprint anything appearing in these pages free of charge and without writing to the WHO for permission.’^52^ Should editors want to use the stories as a starting point for more in-depth features, the PIO also advertised the availability of additional text and illustrations upon request.

The key feature of such publications was their focus on photographs. In 1952, 200 photographs were distributed per month in response to external requests, and escalating demand necessitated that the WHO acquire its own photographic laboratory the following year. By 1957, 40,000 photographic prints were prepared for distribution, and in 1966 this number exceeded 50,000.^53^ Although photographic production was centralized in Geneva, distribution and the acquisition of new material relied on the assistance of information officers throughout the regionalized structure. The Third Annual Report to the Regional Committee for South East Asia recorded photography’s prominence among public information activities, describing interest from newspapers, radio stations, health officials, and UN and welfare organizations.^54^ In 1956 photographic distribution was facilitated by supplying all regional information officers with a stock of reproductions from the *Newsletter*.^55^

The *Newsletter* and *World Health* were visually lavish, and many issues included stories told primarily through photographs. The semi-regular ‘WHO in action’ series illustrated health topics with a selection of photographs, collated from around the world. In other examples, ‘SEEN by photographers of World Health around the globe’ depicted malaria eradication with views selected from over 1,000 photographs,^56^ while ‘Leprosy demystified’ distilled key images from some 25,000 photographs taken by Pierre-Andre Pittet.^57^ Although offered as visual evidence of the WHO in action, such stories frequently relied on broader narratives about the suffering of those affected by disease and how they were helped. To appreciate how these stories fitted in with the broader demands of public information, it is necessary to go beyond and behind the published photographs to explore the organizational culture responsible for their compilation and selection.

## As seen through the eyes of the WHO

By the mid twentieth century, citizens around the world were inundated with visual messages about health and humanitarianism. Humanitarian organizations had increasingly circulated imagery in pamphlets, newsletters, and publications since the beginning of the twentieth century, with the volume intensifying in the interwar years.^58^ Visual messages about health appeared in governmentally produced education pamphlets, exhibitions, and films. Health was also covered by illustrated magazines, where photographs were frequently *the* story. *Life* magazine’s photographers helped to visualize the unfamiliar, introducing readers to new medical procedures as well as conditions in far corners of the globe. In conjunction with photography’s ability to display people, events, and artefacts with apparent realism, there was now a trend towards a more emotional, documentary photography. The Magnum collective, founded by a group of European photographers who were ambivalent to traditional power structures, emphasized ordinary people and everyday experiences through their work.^59^ The 1948 photo essay ‘People are people the world over’, by Robert Capa, published across twelve issues of the *Ladies’ Home Journal*, sought to document the commonalities in human existence.^60^ It laid the groundwork for subsequent photo essays and exhibitions such as the 1955 *Family of Man* exhibition curated by Edward Steichen.^61^ *Family of Man* was based on the idea that photography had a unique ability to explain people to one another, demonstrated through a pictorial focus on significant moments of human existence such as birth, love, work, and death. Subsequently touring the world and proving extremely popular, the exhibition provided an optimistic view of life and a message of hope and brotherhood. As Jean-Claude Gautrand argues, it represented the triumph of humanist photography characterized by sensitivity to the ‘simple joys of life, an empathy for people in the street, caught in action’.^62^

The UN and its specialized agencies shared the view that photographs could help to foster attention and understanding across geographical, cultural, and linguistic barriers. Public information officials were able to discuss planning and policy, and to share common problems at bodies such as the Consultative Committee for Public Information and the United Nations Film Board.^63^ However, each agency honed its own systems and styles in response to individual requirements. One of UNESCO’s earliest missions was a photographic report on the needs of children in Poland, Greece, Italy, Hungary, and Austria, but, like the WHO, it also published a public-oriented magazine, the *UNESCO Courier*, which included arresting photography.^64^ A major function of UNICEF’s information policy was fundraising, and the agency relied on photographs as evidence of its work by instructing commercial photographers to capture the *absence* of disease and hunger, as well as poignant photographs of mothers and children that were frequently repeated to garner public support for its initiatives.^65^

The WHO’s photographs were initially contributed by the agency’s own field project workers, but were ultimately deemed to lack professional quality because they were generally taken by people ‘unfamiliar with the media of information’.^66^ In May 1950 a visual media expert was assigned to arrange photographic missions treating the WHO’s worldwide activities, with the first professional mission completed in 1951.^67^ The WHO embedded photographers within the agency: the Frenchman Didier Henrioud and the Hungarian Tibor Farkas coordinated efforts at WHO headquarters as well as contributing their own reports, while Ajaib Kochar (born in what had become Pakistan) worked as an information officer at the Regional Office for South East Asia. However, the agency relied predominantly on well-regarded photographic agencies such as Magnum, and eminent individuals including the American Homer Page, the French photographers Marc Riboud, Jean Philippe Charbonnier, and Eric Schwab, and the Swiss photographer Jean Mohr. The contributions of internationally known professionals addressed the difficulty of obtaining vivid first-hand accounts of field activities from technical staff, and lent the *Newsletter* and *World Health* cachet and authority. Because of their practical experience, these photographers were also able to operate in difficult conditions and deal with taxing travel commitments.

Photographic narratives were built up in response to the input of many individuals. Once each mission was authorized, photographers were supplied with background information on the countries and the topics in question, and met with regional WHO officials as well as specialists and heads of facilities. Guidelines about places to visit and what to photograph were flexible, since it was acknowledged that missions would have to adapt to unforeseen and unavoidable circumstances. Some photographers were able to follow the human drama as it unfolded. Erling Mandelmann’s detailed mission report on Sweden’s drug problem described what he himself saw, including ‘a young man take [*sic*] his injection openly on a bank – even [though] addicts are chased strongly by the police’, and ‘objects that the police has [*sic*] found under raids … from Opium and Cannabis-Cakes to Amphetamine’.^68^ Mandelmann traced the addicts around the city as they evaded the police.

In other cases, however, very specific instructions were supplied. For a mission on public health in the English city of Liverpool, a list of preferred shots and topics was provided to the photographer Spooner (no first name given), who was asked to capture the ‘little-known, silent, invariably unseen and un-lauded’ health services of the city, and show the medical officer of health as an ‘unseen, unheard of “guardian angel” of health’. Under the heading ‘Human Contact’, the script requested a ‘possibly symbolic shot showing personal contact between Professor Semple, MOH [Medical Officer of Health], and people he is forced to prosecute – on steps of law courts (imposing building of grimy black Portland) after prosecution, if such a case comes up’.^69^ In another case, Josef Breitenback’s mission report on cancer in Japan suggests that he too was given specific requirements because he wrote that he had been unable to photograph former patients at home: ‘Such a thing takes weeks of preparation and talks, more so in Japan than elsewhere to obtain permission.’ Breitenback instead offered amateur photographs of a doctor posed with patients.^70^ Comprising dozens, and sometimes hundreds, of individual shots, each mission returned a range of views that helped to set the scene, as well as showing actors and equipment.

**Figure 1:**
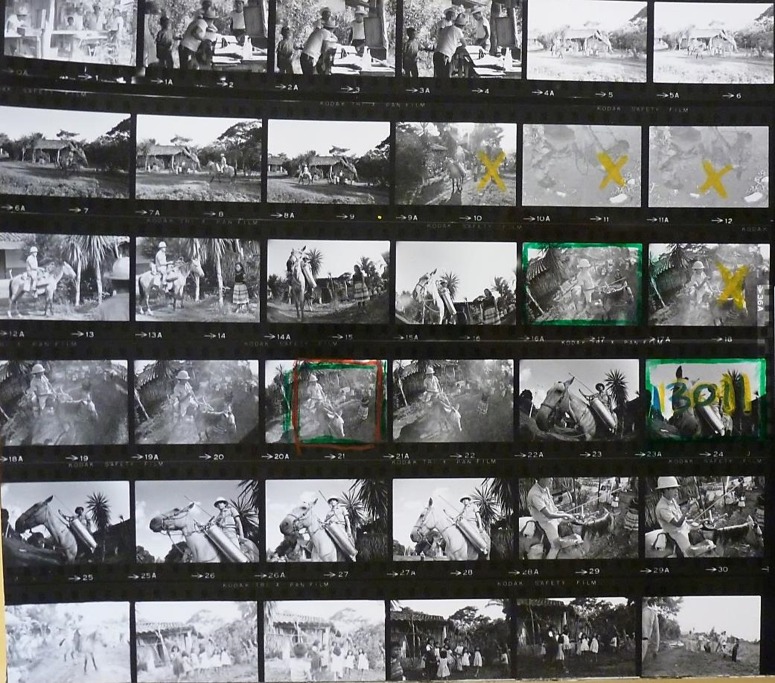
Contact sheet from Peter Larson, Malaria, Mexico, 1968. Photographic Archives, Sub-fonds Photographers, WHO Archives. Copyright WHO.^73^

**Figure 2:**
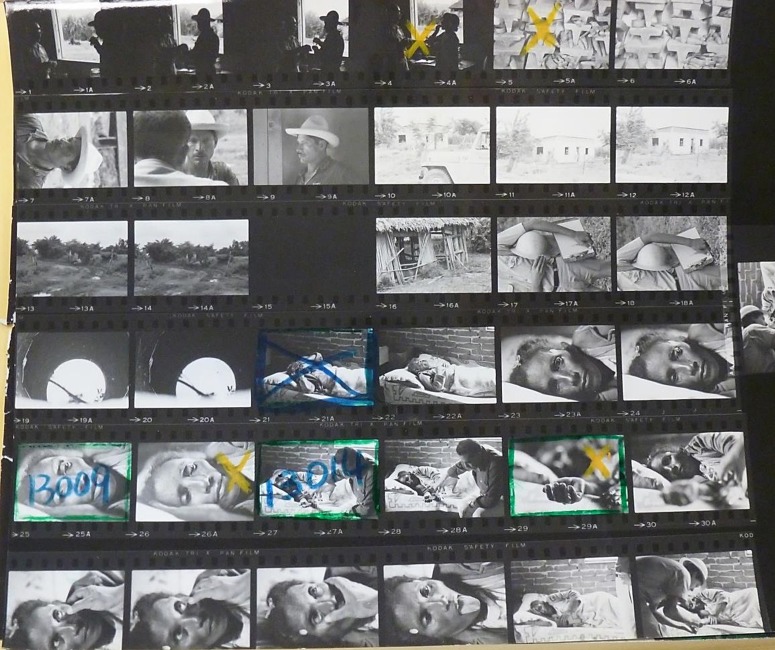
Contact Sheet from Peter Larson, Malaria, Mexico, 1968. Photographic Archives, Sub-fonds Photographers, WHO Archives. Copyright WHO.^76^

But, as Amy Lyford and Carol Payne caution, the practice of photojournalism should not be glorified at the expense of the deliberation and complexity of the wider picturing process.^71^ All the film shot on location was assembled into contact sheets and returned to the WHO.^72^ The contact sheets enabled PIO officials to inspect and compare the work; using crosses, lines, and circles in thick pencil they identified features which were unsuitable, areas to be cropped, and the shots approved for publication. For the historian this intervention provides insights into how visual narratives were built up and how the stipulations of public information were transferred via the camera and onto the page. Figures 1 and 2, from Peter Larson’s 1968 photo mission on malaria in Mexico (which comprised twenty-five contact sheets in total), include photographs of patients, doctors, spray teams, equipment, scenes of village life, dwellings, and the local landscape. Figure 1 shows the malaria sprayman captured in various arrangements, including interacting with villagers and as seen from their perspective. The latter (marked ‘13011’ on this sheet), ultimately selected for publication, portrayed the sprayman as a warrior, posed in a style emulating a knight of old and thus implicitly endorsing the WHO’s characterization of the malaria programme as a war on an ancient foe. Larson’s photograph fitted into a trope established since Eric Schwab’s 1958 photo mission to Mexico, when the suggested text argued that ‘this is WAR, but to save lives, not to sacrifice them, and it calls for the thoroughness of military organization in planning and in logistics … High-ranking military officers direct the campaign from an operational headquarters, from which they deploy their spray-teams as [Fn fn74]though they were combat troops. “Supreme Headquarters” is WHO’s Regional Office’.^74^ Figure 2 displays the similar diligence applied to create a haunting photograph of one of the victims of this war. The contact sheet carries shots of the patient’s alert and piercing gaze, but the crosses show how these were rejected in favour of a sleepier vision of despair and emptiness. Her hand, extended limply out towards the viewer, was preferred to more detailed shots of her examination. The text painted her as ‘the face of malaria’, the material impact of an ‘ancient curse’ which brought ‘delirium, death and economic ruin’.^75^

There is little evidence of consistent attempts to enhance such views by altering them mechanically before publication. Although David and Rodogno cite an example in relation to a story about premature babies, a comparison of contact sheets with published images suggests that this was not an everyday practice, perhaps used when no other alternative was available or in response to specific [Fn fn77]time pressures.^77^ Published photographs tended to appear faithful to the originals, albeit cropped and organized in relation to text and other images. The WHO wanted the photographs to speak for themselves as an unembellished and authoritative record of health ‘seen through the eyes of the WHO’. Yet, while little mechanical manipulation took place, the view was shaped in diverse ways by the instructions given to photographers, by the guides who accompanied them, by the people they photographed, and by the selection process after the films had been returned to the WHO. Indeed, as seen above, the captions and accompanying texts instructed the viewer in how to approach the images. The PIO was further complicit in skewing the picture by featuring photographs which emphasized good practice, or by including examples where programmes were fully operational. Ultimately, the process was geared towards the creation of a carefully staged drama rather than snapshots from the front lines of health.

**Figure 3:**
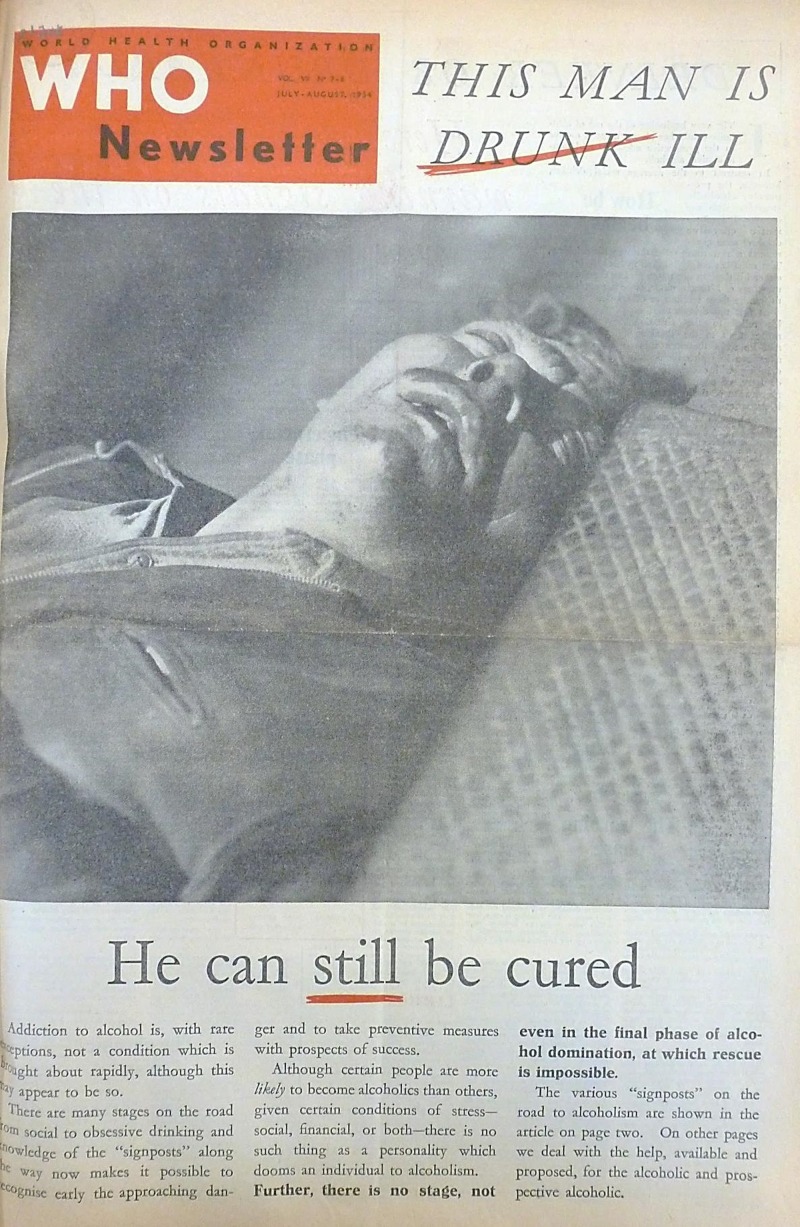
*WHO Newsletter*, July–August 1954, front cover. Copyright WHO.^78^

**Figure 4:**
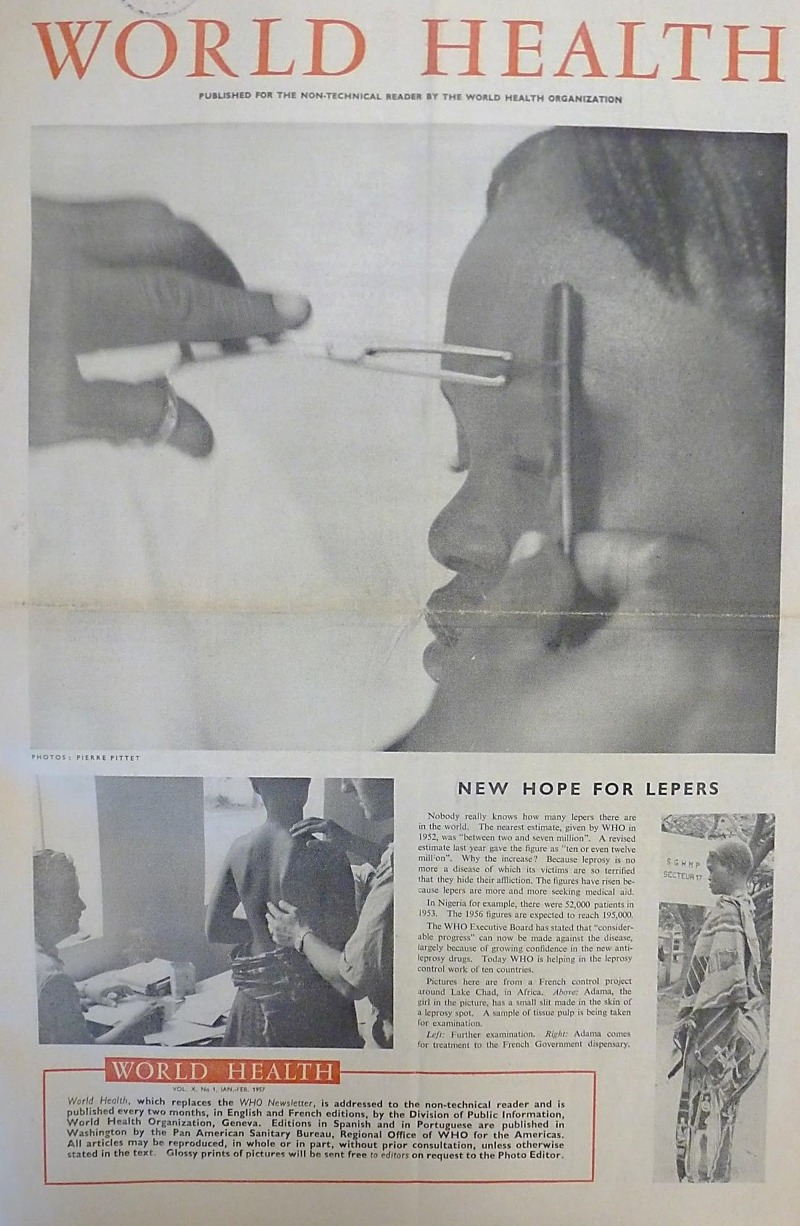
*World Health*, January–February 1957, front cover. Copyright WHO.^80^

Though international health was played out across the world by including human faces in all manner of stories, often rather simple techniques were used to direct the reader’s attention and generate both [Fn fn79]understanding and empathy. Miserable faces underscored the need to solve problems; damaged ones stressed the urgency and magnitude of problems. The cover image chosen for a *Newsletter* issue on alcoholism pictured a man lying incapacitated on the ground (Figure 3). He looked tormented, with his mouth partially open and shadows playing across his deeply furrowed brow. The image was tilted at an unusual angle, stressing the intoxicating effect of alcohol, and life spiralling out of control.^79^ Such constructions were meant to make viewers feel for the plight of the ‘victim’ and pay attention to the message. But notions of villainy, victimhood, and deliverance were all combined in this image. In other stories the damage wrought by leprosy, yaws, and smallpox was plain to see, but the WHO also did not shy away from presenting the realities of medical procedures in their ability to prevent and cure this damage. The lead [Fn fn81]story in the first issue of *World Health*, January–February 1957 (Figure 4), carried a close-up photograph of a girl called Adama, her eyes closed, as a small incision was made to collect a tissue sample to test for leprosy. At first glance, the most visible and disturbing sight is the implement poised to break Adama’s skin, although this is intended to assure the reader that timely action has saved her from further disfigurement, since further down the page she is shown on her way to receive treatment.

**Figure 5:**
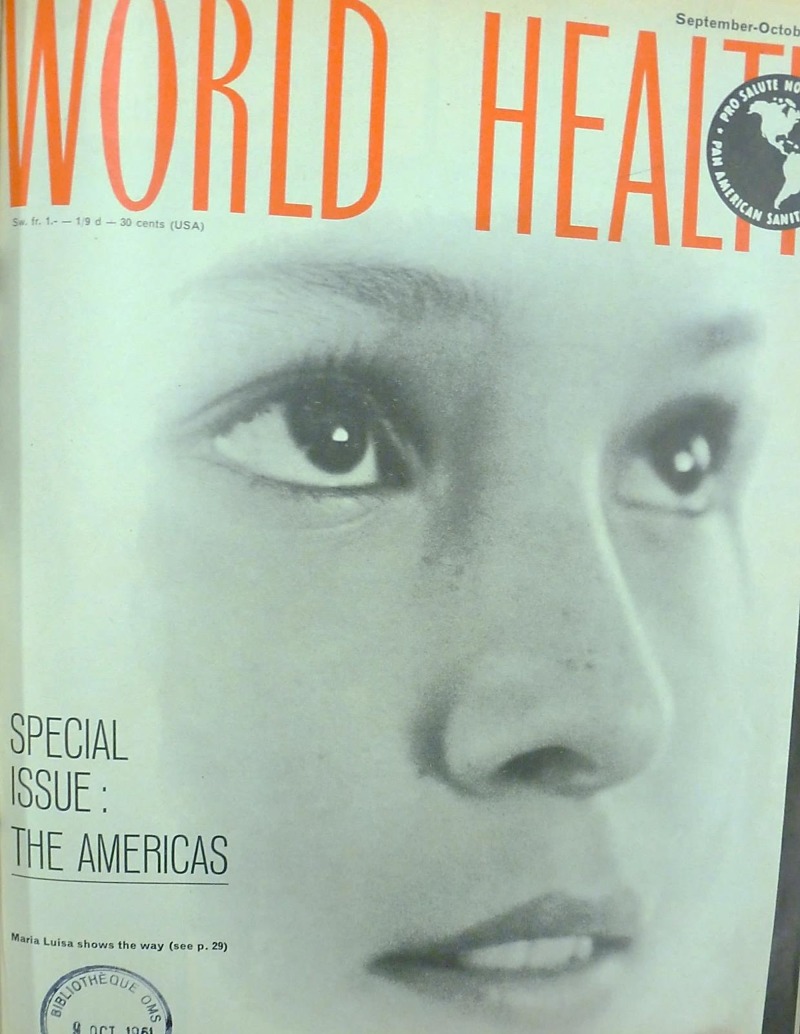
*World Health*, special issue: ‘The Americas’, September–October 1961. Copyright WHO.^81^

In contrast, happy and hopeful faces were used to show problems being resolved, to inspire further action, and to approve the work of the WHO. But this went beyond ‘before and after’ narratives that showed damaged faces and healed ones following treatment. The innocent faces of children, or the arresting faces of young women, drawn from countries around the world were used to capture the viewer’s interest, but this was not always done to inspire pity, concern, or relief. Optimism was also a strong feature: Maria Luisa ‘showed the way’ on the cover of a 1961 special issue [Fn fn82]on health in the Americas (Figure 5). In looking upwards and to a fixed point in the distance, she appears to suggest that, although still some way off, the way forward represented a bright and alluring prospect. Indeed, one might read her expression as anywhere between dreamlike and fixated on this idea of hope. Although her name is given, and facial features and expression are clearly visible, the blurring of the edges of Maria Luisa’s face omits contextualizing information such as hairstyle, clothing, or location, giving the impression of an every-person. This cover photograph testifies to the WHO’s preoccupation with human interest, but also its desire to move beyond the medicalized gaze and on to universally felt emotions of determination, hope, and happiness.

**Figure 6:**
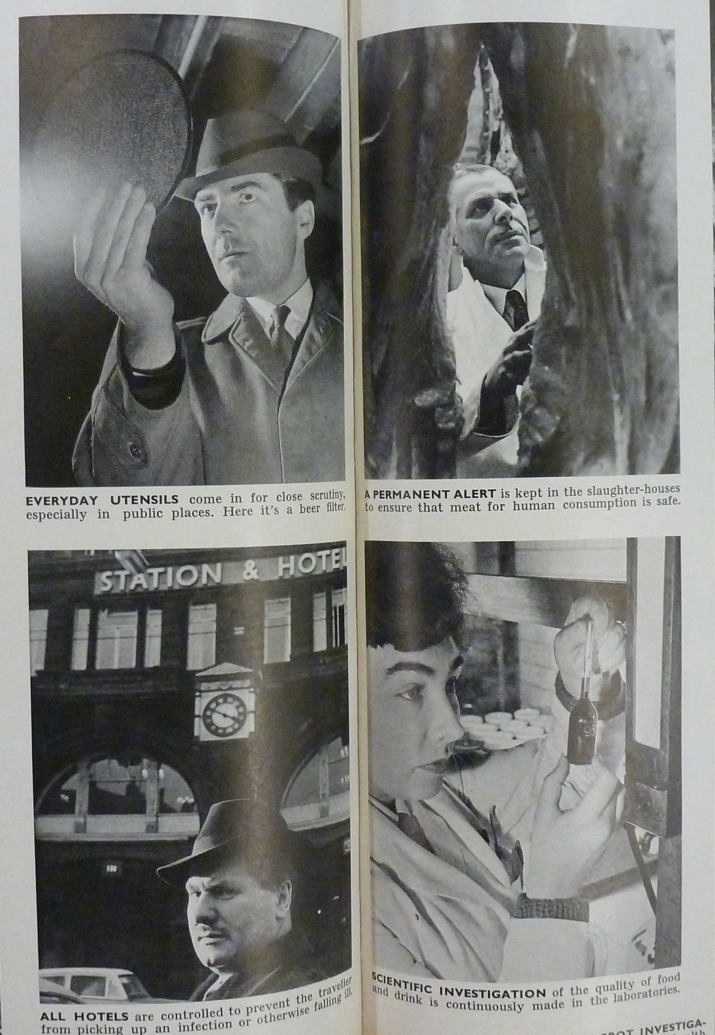
Detail from ‘The unknown guardian angels have faces’ photo story, *World Health*, September–October 1963, pp. 48–9. Copyright WHO.^86^

While the tale of victory has been identified as an important pillar of the WHO’s visual politics,^82^ it was equally important to visualize the work undertaken to achieve this in photo stories that described searching, hunting, and scouring. The images were intended to support the notion that all possible efforts were being marshalled to combat and overcome the causes of ill health. Such efforts could be initiated, for instance, by innovative technology and scientific breakthroughs, with reportage taking place in the laboratory and the operating theatre, where the actors searched for answers and pursued microscopic enemies. In the 1963 feature ‘The unknown guardian angels have faces’ (Figure 6), the photographs showed public health officials examining hotels, as well as utensils and foodstuffs. Again, the viewer is invited to scrutinize their faces, and, while comparatively stoic, their expressions of concentration underscored the diligence and expertise necessary to prevent people falling ill.^83^ *World Health* January–February 1962 carried a story of a Hong Kong opium hunt, in which the photographs, by the Hungarian Paul Almasy, depicted policemen and specially trained dogs searching the streets for drug peddlers. One even showed a pursuit in progress across the roofs of the town as smokers escaped through a window.^84^ But the same point was made in relation to the dedication to the betterment of humankind which was held to thrive in every person, regardless of their location or background. The impression of a pursuit added drama and constituted an important narrative stepping stone in relation to the WHO’s ‘before and after’ photographs, which showed problems and resolutions. But such photo stories also added weight to the implication that people should maintain their vigilance in the face of threats to public health.

Although human actors were privileged in the drama of international health, place and location played an important role in structuring and staging the photographic performance. In depicting remote rural areas, the magazines asked readers to consider the difficulties of staying healthy in inaccessible places rarely reached by the modern world. However, such landscapes were also presented as stunningly beautiful in their loneliness, and this aspect was regularly emphasized in *World Health*. They were used to tempt the reader’s attention to these mysterious places (articles commonly began with long descriptions about unusual fauna and flora) and add a sense of adventure to delivering health in these areas. They were similarly constructed with an eye to conveying the ‘right’ message, even when this entailed re-enactment: when photographing a ‘stranded’ jeep to illustrate the difficulties of communication, a group of local children rushed to the rescue, believing the jeep to be in genuine difficulty.^85^ In contrast, while cities generally benefitted from better access to health services, they were paradoxically represented as highly dangerous places. Hazards lurked around many corners, in the shape of traffic accidents and inadequate sanitation, but also in regard to modern living with addiction to alcohol and drugs, as seen in the example above. Implicit in the WHO’s publications was the message that, although people were people the world over, they were also specifically located and subject to the problems associated with different environments.

It is beyond the scope of this article to detail the myriad ways in which location was used, but it is worth considering some examples to demonstrate that a variety of staging devices were used to draw readers to certain conclusions. Whether rural or urban, the family home was presented as being at the heart of human health. It was a setting frequently used to display the mother and child relationship emphasized by other organizations such as UNICEF. Workplaces, often depicting agricultural or factory work, were shown both as a site of potential danger from industrial accidents or respiratory disorders and also as an element in the narrative of resolution, whereby returning to work signified productive life recommencing after[Fn fn87] illness had successfully been overcome. The setting of the hospital or the laboratory was used in diverse ways, and conformed to the wider representation of places where healing and discovery took place. Finally, the administrative buildings of the WHO were shown as orderly, tranquil, and purposeful centres where matters were resolved and a healthier future mapped out.

Sometimes the WHO persisted with a particular image which, despite inaccuracies, was thought to capture the public imagination. This was the case in establishing the idea of a military operation against malaria. Following Eric Schwab’s 1958 photo story on the Mexican eradication effort, the WHO was subsequently advised that the participation of army personnel was solely under Ministry of Public Health control, and was asked to avoid any further confusion.^87^ However, as Larson’s photographs used some years later demonstrate, the WHO continued to convey the impression that the Mexican military was involved. Certain photographs were reused in later issues and other publications, sometimes in connection with topics they were not originally intended to illustrate, albeit cropped differently or accompanied by reworked captions. This emphasizes that photography’s value for public information purposes lay not simply in recording situations realistically and keeping the public informed, but also in stimulating and directing popular opinion. A good picture was perennially useful, and by the late 1960s the WHO’s central photo library contained more than 15,000 registered negatives. The WHO believed it to be the world’s most extensive collection of pictures on the situation of international health, capable of illustrating health activities and achievements in almost any area of the world.^88^ Different influences and specific styles were evident in every photograph, but the WHO ultimately preferred those which were eye-catching, and which supported a narrative where ‘scientific accuracy’ was arguably relegated behind ‘attractive and popular’ in the procurement and selection process. The photographs were required to do much more than make the WHO’s work visible, and the photographer was less a witness and more a storyteller.

## The problems of public information

The WHO periodically evaluated public information work, but success was difficult to determine explicitly. The circulation of the *Newsletter* and then *World Health* grew throughout the 1950s and 1960s, and the supply of other written and visual material was commonly exhausted within months of release. These accomplishments were enthusiastically announced in annual reports, although it was acknowledged that numbers alone did not represent a valid criterion for assessing the true effectiveness of the work, which was cumulative and dependent on sustained efforts.^89^ Nevertheless, the WHO increasingly asked whether public information expenditure represented good value, and what more could be done to meet its constitutional obligations. It was probably sensitized to these issues because of early failures and missed opportunities. Although the PIO was established to inform the greatest number of people about the agency’s work, it was ironically unable to satisfy the initial levels of interest in the agency. In 1947 the Interim Commission highlighted the small provision for PIO staff compared with other UN agencies (two officials compared with twenty-four at the Food and Agriculture Organization and fifteen at UNESCO), and advised creating two more permanent posts.^90^ But subsequent reports noted that not only was the PIO unable to satisfy demand, but it also had to turn down requests for news items and photographs from popular science magazines, general health and paramedical publications, yearbooks, encyclopaedias, and handbooks on international organizations. The annual report for 1950 stressed that interest in the work of the WHO was ‘almost everywhere greater than are the possibilities of supplying information about it’.^91^ Only in 1953 was it possible to fulfil the majority of requests. This inability to capitalize on early interest also fuelled the conviction that the WHO could not hope to spread evidence of its existence independently, but needed to rely on other media outlets to cover the WHO on its behalf.

With the limited resources at its disposal the WHO was similarly unable to provide translations to meet demand. It therefore depended on local efforts: in 1953 the Regional Committee for the Western Pacific recommended that each member state take steps: first, to reproduce and distribute information concerning the work of the WHO ‘on the widest possible scale’; second, to establish a liaison service; and third, to teach the aims and activities of the WHO to schoolchildren.^92^ Concluding that public information activities should be intensified, the Eighth World Health Assembly, the main decision-making body of the WHO, made a similar request to member governments to ask for cooperation in making the WHO’s work, limitations, and challenges widely known.^93^ As new nations joined the UN in the late 1950s they were approached by the WHO, which emphasized that they should allocate all necessary resources to help realize the objectives of the agency.

As mentioned above, the WHO hoped to attract external coverage through its magazines, and later in the 1960s the agency also succeeded in placing its photographs in United Press International’s EUROPIX service, which served Austria, Belgium, France, the Federal Republic of Germany, Hungary, Italy, Netherlands, Peru, Scandinavia, Spain, Turkey, the United Arab Republic, and the United States of America.^94^ These efforts met with some success: a report on public information projects undertaken between November 1953 and June 1954 recorded that the popular French magazine *Elle*, and other illustrated periodicals in Switzerland, the UK, and Germany, had devoted long stories to the WHO. The regional offices worked to inspire international coverage by arranging for material to be placed in local picture magazines.^95^ In 1955 it was reported that a ‘popular health magazine’ with a circulation of 300,000 had reprinted the entire July–August 1954 issue of the *Newsletter*.^96^ In 1964 the Oxford Committee for Famine Relief (OXFAM) used WHO photographs in its *Partners in Progress* exhibition, and a dozen magazines published the ‘Mask of meningitis’ photograph series which illustrated a WHO-assisted project on cerebrospinal meningitis control in Niger.^97^

*Life* and *Life International* magazines used WHO photographs in several stories about the agency, such as the ten-page article in August 1958 that featured stories on ‘A polio boy on his feet’, ‘Malaria menace tops all others’, and ‘A world war on disease’.^98^ Although copyright freedom meant that there was no need to attribute the accompanying images to the WHO, most would have been familiar to regular readers of the *Newsletter*. Coverage of the WHO in *The Rotarian*, the official magazine of Rotary International, was more embellished. The article ‘500 million children I can’t forget’ emphasized the WHO as a world good, outlining the health problems facing children globally, and the WHO’s role in solving them. The article featured Eric Schwab’s photographs of Ede Nwaegbo before and after his treatment for yaws. Ede’s story was relayed in the following words:

A year or so ago he got yaws … and his face looked like this (left). Even his mother could barely stand the sight of him. Then a WHO team came along and gave him a shot of penicillin. Ten days later he looked like this (right). Today, he’s as round, smooth, and lovable as any 5-year-old. His new lease on life cost the equivalent of one U.S. Dollar.

The article concluded by proclaiming, ‘this work is monumental and well worth the millions of dollars that WHO and UNICEF expend’.^99^ Whether the WHO was pleased with such brazen positivity, or feared it as more akin to propaganda, is not recorded in the annual reports. But, like the UN, the WHO probably considered that coverage of its activities in well-regarded and prestigious outlets helped to enhance its reputation and promote the newsworthy aspects of the agency.^100^

Occasionally, however, the use of WHO photographs by external publications caused controversy. An illustrative case occurred after a 1970 shoot on cancer in Kenya. Some months after Paul Almasy’s photographs were published in *World Health*, the photo editor, Tibor Farkas, received a letter from the medical centre involved in the reportage. The director said that he had been shocked to see his photographs reproduced in the *Medical Tribune*, because they presented ‘a confused and completely erroneous picture of our aflatoxin and liver cancer programme’ which could, he claimed, have political and social repercussions.^101^ Such was the WHO’s reputation that inclusion of material credited ‘WHO’ could be taken as tacit endorsement even if this was not the intention. In instances like this, however, the WHO had no control over the editorial decisions of independent publications and could not ask each story to be sent to the agency to be proofed. Distributing material on a large scale and encouraging outside publication was a key public information strategy.

In other instances the WHO sternly protected its identity and reputation. This is evident in the Brando case, and in the efforts to guard against the unauthorized use of the WHO name and emblem.^102^ Its reports might appear outward-looking, but in certain cases the WHO remained extremely cautious about its global representation. Although the agency would have agreed with the UN’s broad ideas and goals regarding public information, it was more reticent regarding the specific ways through which these aims could be achieved. Cinema’s popularity made it a natural area of interest for the UN, and the film board was envisaged as a body which would not only make its own newsreels and documentaries but also stimulate outside film production on the activities of the UN and its agencies.^103^ Jean Benoit-Levy, head of UN film activities, and Benjamin Cohen believed that, if studios could be convinced to feature the UN, this would help to condition audiences to accept the organization as part of their daily lives and associate it favourably with high purposes. If successful, this would garner millions of dollars’ worth of publicity for the price of one man’s time and wages.^104^ The former head of the UNESCO Film Section in Paris, Mogens Skot-Hansen, was selected to supply freelance writers with story ideas and material, and information on the UN and its agencies.^105^ But at a meeting in July 1951, Joseph Handler, the head of the WHO’s PIO, reported that, although Skot-Hansen had performed an excellent job on behalf of the UN, judgement and sometimes restraint should still be exercised in approving Hollywood scripts containing UN references.^106^ This attitude helps explain why the Brando film stalled, despite the fact that it represented precisely the kind of opportunity that Skot-Hansen had been tasked with securing.

If public information was to have any value and be acknowledged as truthful and authoritative, the agency needed to safeguard its reputation by preventing the unauthorized use of its image. A film screened before WHO officials in January 1963 was rejected, owing to the unsuitable treatment of venereal disease and prostitution. It was decided that the WHO could not be associated with the film in any way, and that references to the WHO name, buildings, and activities should be removed. Yet an edited version of the same film was again deemed unacceptable because it seemed to suggest that the characters were connected with the WHO, a suggestion reinforced by the fact that the WHO was mentioned in the acknowledgements and commentary. A recall and edit was promised, and the existing footage was constantly monitored to ascertain whether any illicit copies remained in circulation. The agency even used its legal office to contact critics who had reviewed the film for newspapers.^107^ This episode again demonstrates the frequently fine line in the demands placed on public information officials as they sought to promote their activities but guard against questionable usage of the WHO’s association. But it also suggests the presence of companies and individuals that accepted the credibility and authority of the WHO (as stated in the agency’s own public information materials), but used this connection in ways that could be inappropriate or controversial.

Judging the effect of public information work on the intended audience was harder. The records do not suggest that a comprehensive answer was ever obtained, although the WHO had a reasonably good idea of whom it wanted to target and whom it was reaching, recording the names and addresses of subscribers to its publications. The PIO initially employed a press-cutting service, covering Switzerland, ‘certain areas of Europe’, and ‘a few non-European centres’, which delivered some 800 to 1,000 clippings from newspapers and periodicals.^108^ In 1968 press cuttings received from ‘all parts of the world’ indicated widespread interest in that year’s World Health Assembly, as well as topics including cancer, cardiovascular diseases, smallpox eradication, the growing accident rate, family planning, and the shortage of medical and nursing personnel.^109^ But the same sources also revealed disappointments, such as poor press coverage of the 1959 world malaria eradication campaign.^110^ Internal reports suggest that the WHO was keen to learn from such failures and renegotiate its appeal: the ‘constant problem’ of interesting the press in positive health achievements rather than sensational or controversial topics led the PIO to change the presentation of certain press releases by including more background information and lively examples. It was subsequently noted that the strategy resulted in increased interest, although quite how this was demonstrated is unclear.^111^ It also remained difficult to estimate the size of the audience reached by WHO information, and even harder to assess the extent to which public attitudes were changed.

There are also very few opportunities to assess how the WHO’s photographs were viewed by the public at large, although a photography competition published in *World Health* of May 1971 provides some telling insights. The front cover featured nineteen photographs with a banner title encouraging readers to ‘Look carefully at this cover: it may win you a prize’. They were asked to identify the health activity illustrated in each of the photographs, with bonus points given for naming the country. The top prizes ranged from a camera, transistor radio, and wristwatch down to WHO albums, keychains, and subscriptions to *World Health*. The competition required close scrutiny of the photographs, as well as a familiarity with the topics and stylistic conventions in the publication. Although the winners came close, none of the entrants correctly identified all nineteen photographs. This suggests that, while people were interested in the photos and found them visually intriguing, they had not taken note of the particular medical procedures or the geographic location. Since readers were educated to see emotions and shared problems, photographs were less useful in engaging people as informative indicators about particular health situations in specific countries.

## Conclusions

This article has sought to understand the formulation of the WHO’s public information programmes and its impact on subsequent visual campaigns. The success of the WHO’s mission was thought to depend on its ability to communicate; as a multifaceted international organization with multiple regional variations and programmes to consider, the new agency aimed to establish its identity and authority, and to build a rapport with the world public. In doing so, its communications went beyond keeping the public conversant with the international health situation and sought to create enthusiasm and interest in the WHO’s activities. As the UN’s incursions into Hollywood show, this was a key period in working international organizations into the everyday lives of citizens around the world. However, in an increasingly crowded mass media landscape, many organizations competed for public attention.

Control of the WHO’s identity was equally essential, and by the time that the Brando film proposal was being seriously discussed the organization was increasingly nervous about relinquishing control over its messages, and about potential damage to its carefully crafted public image. While the agency competed for attention it did not do so indiscriminately. Health was of interest to all peoples, but required sensitive treatment if new measures were to have any chance of success. The importance of this can be seen in the complementary drive to monitor the performance of campaigns and evaluate impact, as well as in the increasing reliance on media professionals rather than field workers with an amateur interest in the subject.

This article has nevertheless shown how and where the boundaries were pushed. Photography was emotional; mission reports and correspondence with photographers demonstrates that photo stories were the result of a painstaking process and the work of many officials. Public information was intended to build up a ‘world health-conscious’, but it is unclear whether those responsible were concerned that their efforts created visual vocabularies which were stereotyped, or whether there was an awareness of contemporary phenomena akin to ‘compassion fatigue’.^112^ In evaluating programmes, the WHO’s records are ultimately silent on these issues, even though the annual reports do record instances where officials were dissatisfied by the response to certain campaigns. It is quite possible that some adverse aspects were not fully recognized until later on, when more serious attempts were made to evaluate public information work qualitatively. For instance, media enquiries around the delivery of primary health care revealed how inured people had become to promises of betterment, but the reader experience is, on the whole, difficult to retrace.

While in the 1950s and 1960s the WHO released material in the hope that it would be sufficiently attractive to be picked up, it subsequently acknowledged that a vertical approach to public information was insufficient, and that local communities and journalists needed to be involved in the process. Information was not necessarily imparted correctly or understood automatically as a result of press releases.^113^ The WHO also recognized that communications technology worked at different pitches around the world, and that a one-size-fits-all approach was ineffective. New approaches and technologies led to questioning of how to best publicize the WHO’s work and address criticisms; the popularity of television had, for instance, meant the decline of many mass-circulation illustrated magazines.^114^ Image management took on renewed importance in the late 1970s and 1980s, with greater questioning of the WHO’s authority and capabilities, especially when it experienced financial instability as a result of zero nominal growth and the failure of some countries to pay their full assessed contributions.^115^ As a result, public information activities were reduced between 1976 and 1979, with some elements eliminated altogether: exhibitions would only be issued if funding was found through extra-budgetary resources. The complete curtailment of the free distribution of photographs marked a new embattled era of media engagement and crafting of the WHO’s public persona.^116^

## Footnotes

1 WHO Archives, Geneva, General Aspects of Film Production, Policy, Distribution, V6/158/1, Brando Film Group Correspondence.

2 WHO Archives, General Aspects of Film Production, Policy, Distribution, V 6/158/1, 8 March 1956, Brando Film Group Letter.

3 ‘Marlon Brando wants to make film on UN’, *Sarasota Journal*, 22 July 1959.

4 Bosley Crowther, ‘“The ugly American” opens: Marlon Brando stars as the U.S. diplomat’, *New York Times*, 12 April 1963, http://www.nytimes.com/movie/review?res=9403E7DD103CE63ABC4A52DFB2668388679EDE (consulted 20 October 2017).

5

AlleyneMark, Global lies? Propaganda, the UN, and world order, Basingstoke: Palgrave Macmillan, 2003, p. 23 .

6

AllbesonTom, ‘Photographic diplomacy in the postwar world: UNESCO and the conception of photography as a universal language, 1946–56’, Modern Intellectual History, 12, 2, 2015, pp. 389–415 ; DruickZoë, ‘UNESCO, film, and education: mediating postwar paradigms of communications’, in Charles Acland and Haidee Wasson, eds., Useful cinema, Durham, NC: Duke University Press, 2011 .

7 Allbeson, ‘Photographic diplomacy’, p. 389.

8

BriggsLaura, ‘Mother, child, race, nation: the visual iconography of rescue and the politics of transnational and transracial adoption’, Gender and History, 15, 2, 2003, pp. 191–193 .

9

PackardRandall, A history of global health: interventions into the lives of other peoples, Baltimore, MD: Johns Hopkins University Press, 2016, pp. 125–127 .

10

WHO, The first ten years of the World Health Organization, Geneva: WHO, 1958 13577639; WHO, The second ten years of the World Health Organization, Geneva: WHO, 1968 4876794; LitsiosSocrates, The third ten years of the World Health Organization, Geneva: WHO, 2008 ; WHO, The fourth ten years of the World Health Organization, Geneva: WHO, 2011 .

11

OstherrKirsten, ‘Contagion and the boundaries of the visible: the cinema of world health’, Camera Obscura, 17, 2, 2002, pp. 1–38 ; OstherrKirsten, Cinematic prophylaxis: globalization and contagion in the discourse of world health, Durham, NC: Duke University Press, 2005 .

12

HansenBert, Picturing medical progress from Pasteur to polio: a history of mass media, New Brunswick, NJ: Rutgers University Press, 2009, p. 208 .

13

DavidThomas and RodognoDavide, ‘All the world loves a picture: the World Health Organization’s visual politics, 1948–73’, in Heide Fehrenbach and Davide Rodogno, eds., Humanitarian photography: a history, Cambridge: Cambridge University Press, 2015, pp. 223–248 .

14
*Ibid.*, pp. 231–42.

15

WHO, The work of WHO: annual report of the Director-General to the World Health Assembly and to the United Nations, 1950, Geneva: WHO, 1951, p. 71, http://apps.who.int/iris/handle/10665/85609 (consulted 20 October 2017).

16

BorowyIris, Coming to terms with world health: the League of Nations Health Organization, 1921–1946, Oxford: Peter Lang, 2009, pp. 12–13 , 26–9; FarleyJohn, Brock Chisholm, the World Health Organization, and the Cold War, Vancouver: UBC Press, 2008, p. 10 .

17 Farley, *Brock Chisholm*, p. 10.

18 For more on the history and operation of regional offices, see LeeKelley, The World Health Organization, London: Routledge, 2009, pp. 30–34 .

19 Farley, *Brock Chisholm*, p. 111.

20
*Ibid*., pp. 116–17.

21

BrownTheodore, CuetoMarcos, and FeeElizabeth, ‘The World Health Organization and the transition from “international” to “global” public health’, American Journal of Public Health, 96, 1, 2006, p. 65 10.2105/AJPH.2004.050831PMC147043416322464.

22 WHO constitution, Art. 2(r).

23 WHO, *Work of WHO: annual report of the Director-General to the World Health Assembly and to the United Nations, 1951*, Geneva: WHO, 1952, p. 1, http://apps.who.int/iris/handle/10665/85637 (consulted 20 October 2017).

24 WHO Interim Commission, WHO.IC/106, ‘World Health Organization public information’, 1947.

25

PianaFrancesca, ‘Photography, cinema, and the quest for influence: the International Committee of the Red Cross in the wake of the First World War’, in Heide Fehrenbach and Davide Rodogno, eds., Humanitarian photography: a history, Cambridge: Cambridge University Press, 2015, pp. 144–156 .

26

BaughanEmily, ‘“Every citizen of Empire implored to save the Children!” Empire, internationalism and the Save the Children Fund in inter-war Britain’, Historical Research, 86, 231, 2013, pp. 123–124 .

27

TworekHeidi, ‘Peace through truth? The press and moral disarmament through the League of Nations’, Medien & Zeit, 25, 4, 2010, pp. 16–23 .

28

WollaegerMark, Modernism, media, and propaganda: British narrative from 1900 to 1945, Princeton, NJ: Princeton University Press, 2007, pp. 3–13 .

29
*Ibid*., p. 7.

30 Alleyne, *Global lies?*, pp. 4–5.

31

CohenBenjamin, ‘The U.N.’s department of public information’, Public Opinion Quarterly, 10, 2, 1946, p. 146 .

32 Alleyne, *Global lies?*, p. 4.

33 WHO, ‘Study of problems of public information’, 1955, p. 3, http://apps.who.int/iris/handle/10665/104073 (consulted 20 October 2017).

34 WHO Interim Commission, WHO.IC/106, ‘World Health Organization public information’, 1947.

35 WHO, ‘Study of problems of public information’, p. 3.

36 WHO, *First ten years*, p. 445.

37
*Ibid*., pp. 454 and 447.

38 WHO, ‘Study of problems of public information’, p. 2.

39
*Ibid*., p. 3.

40

WHO, The work of WHO: annual report of the Director-General to the World Health Assembly and to the United Nations, 1955, Geneva: WHO, 1956, p. 52, http://apps.who.int/iris/handle/10665/85666 (consulted 20 October 2017).

41

HemmingJames, Mankind against the killers, London: Longmans, Green and Co, 1956, p. 199 .

42 See, for instance, KarpfAnne, Doctoring the media: the reporting of health and medicine, London: Routledge, 1988 .

43 WHO, *First ten years*, p. 447.

44 WHO, *Work of WHO: annual report 1951*, p. 2.

45 WHO, ‘Study on problems of public information’, pp. 13–16.

46

WHO, The work of WHO: annual report of the Director-General to the World Health Assembly and to the United Nations, 1967, Geneva: WHO, 1968, p. 48, http://apps.who.int/iris/handle/10665/85804 (consulted 20 October 2017).

47
*WHO Newsletter*, no. 10, September 1949. Other publications such as the WHO *Chronicle* were acknowledged as the ‘most suitable medium for presenting to professional health workers reliable information on the work of the Organization’. Governing bodies WHA3.A3/81, ‘Reconsideration of the WHO programme of publications’, May 1950.

48 WHO, ‘Study on problems of public information’, p. 4.

49 WHO, ‘Organizational study on publications’, WHO Executive Board Provisional agenda item 2.4(а) ЕЗ25/32, 16 December 1959, p. 40, http://apps.who.int/iris/handle/10665/135456 (consulted 20 October 2017).

50 WHO, ‘Organizational study of publications’, Executive Board E 2/445/3, 7 May 1958.

51 WHO, ‘Organizational study on publications’, December 1959, p. 52; WHO, *The Second Ten Years of the World Health Organization*, p. 292.

52
*WHO Newsletter*, November–December 1951.

53 WHO, Minutes of the fourteenth meeting, Palais des Nations, Geneva, 20 January 1953, Governing bodies EB11, EB 11/Min/14 Rev. 1; *Annual report of the Director-General to the World Health Assembly and to the United Nations, 1957*, Geneva: WHO, 1958, http://apps.who.int/iris/handle/10665/88756 (consulted 13 November 2017); *Annual report of the Director-General to the World Health Assembly and to the United Nations, 1965*, Geneva: WHO, 1966, http://apps.who.int/iris/handle/10665/89361 (consulted 13 November 2017).

54 WHO, SEA/RC4/2, Third annual report of the Regional Director to the Regional Committee for South East Asia, New Delhi, 30 August 1951.

55

WHO, The work of WHO: annual report of the Director-General to the World Health Assembly and to the United Nations, 1956, Geneva: WHO, 1957, p. 47, http://apps.who.int/iris/handle/10665/85682 (consulted 20 October 2017).

56
*World Health*, December 1961.

57
*World Health*, July–August 1960.

58

FehrenbachHeide and RodognoDavide, ‘“A horrific photo of a drowned Syrian child”: humanitarian photography and NGO media strategies in historical perspective’, International Review of the Red Cross, 97, 900, 2015, p. 1123 ; Piana, ‘Photography’, p. 146; Baughan, ‘Every citizen’, pp. 123–4.

59

GoodyearFrank, ‘The face redrawn: Magnum portraits’, in Steven Hoelscher, ed., Reading Magnum: a visual archive of the modern world, Austin, TX: University of Texas Press, 2013, p. 93 .

60

MorrisJohn G., Get the picture: a personal history of photojournalism, Chicago, IL: University of Chicago Press 2002, pp. 113–121 .

61

BerlierMonique, ‘The family of man: reading of an exhibition’, in Bonnie Brennen and Hanno Hardt, eds., Picturing the past: media, history, and photography, Chicago, IL: University of Illinois Press, 1999, p. 209 .

62

GautrandJean-Claude, ‘Looking at others: humanism and neo-realism’, in Michel Frizot, A new history of photography, Cologne: Könemann, 1998, pp. 613–627 .

63 WHO, *First ten years*, p. 453.

64 This was conducted by the photographer SeymourDavid, European photo-correspondent for the New York magazine *This Week*. ‘UNESCO begins photo survey of war-hit nations’, UNESCO Courier, 1, 4, 1948, p. 3 .

65 Briggs, ‘Mother, child, race, nation’, pp. 192–6.

66

WHO, The work of WHO: annual report of the Director-General to the World Health Assembly and to the United Nations, 1953, Geneva: WHO, 1954, p. 59, http://apps.who.int/iris/handle/10665/85650 (consulted 20 October 2017).

67 WHO, *Work of WHO: annual report 1950*, p. 71.

68 WHO Archives, Photographic Archives, Sub-fonds Photographers, 1970, Cardio and Drugs, Sweden, Mandelmann.

69 WHO Archives, Photographic Archives, Sub-fonds Photographers, 1963 Public Health Liverpool, England, 1963, Spooner.

70 WHO Archives, Photographic Archives, Sub-fonds Photographers, 1969, Cancer, Japan, Breitenback, letter to Mr Tibor Farkas, 1 November 1969.

71

LyfordAmy and PayneCarol, ‘Photojournalism, mass media and the politics of spectacle’, Visual Resources, 21, 2, 2005, p. 121 .

72

LubbenKristen, ed., Magnum contact sheets, London: Thames and Hudson, 2011, p. 9 .

73 Reprinted from WHO Archives, Photographic Archives, Sub-fonds Photographers, Peter Larson, Malaria, Mexico, copyright WHO (1968).

74 WHO Archives, Photographic Archives, Sub-fonds Photographers, 1958, Background-Mexico-Malaria-Schwab.

75
*World Health*, April 1968.

76 Reprinted from WHO Archives, Photographic Archives, Sub-fonds Photographers, Peter Larson, Malaria, Mexico, copyright WHO (1968).

77 David and Rodogno, ‘All the world loves a picture’, pp. 228–30.

78 Reprinted from *World Health*, July–August 1954, Copyright WHO (1954).

79
*WHO Newsletter*, July–August 1954.

80 Reprinted from *World Health*, January–February 1957. Copyright WHO (1957).

81 Reprinted from *World Health*, September–October 1961. Copyright WHO (1961).

82 David and Rodogno, ‘All the world loves a picture’, pp. 231–5.

83
*World Health*, September–October 1963.

84
*World Health*, January–February 1962, pp. 18–23.

85
*WHO Newsletter*, February 1952.

86 Reprinted from *World Health*, September–October 1963, pp. 48–9. Copyright WHO (1963).

87 WHO Archives, Photographic Archives, Sub-fonds Photographers, A, Latin America, Health Services, 1958, Homer Page, WHO publication ‘Ten steps forward’, letter, 1 October 1958.

88 WHO, *Second ten years*, pp. 293–4.

89 WHO, *The work of WHO: annual report of the Director-General to the World Health Assembly and to the United Nations, 1948*, Geneva: WHO, 1949, pp. 37–8, http://apps.who.int/iris/handle/10665/85595 (consulted 20 October 2017).

90 WHO Interim Commission, WHO.IC/106, ‘World Health Organization public information’), 1947.

91 WHO, *Work of WHO: annual report 1950*, p. 69; WHO, *The work of WHO: annual report of the Director-General to the World Health Assembly and to the United Nations, 1949*, Geneva: WHO, 1950, p. 76, http://apps.who.int/iris/handle/10665/85603 (consulted 20 October 2017).

92 WHO, WP/RC4/R20, ‘Public information – Regional Committee for the Western Pacific 4th session, Tokyo, 3–8 September 1953’.

93 WHO, A8/AFL/26, ‘Public information activities: resolution proposed by the Delegations of Argentina, Austria, Brazil, Burma, Canada, Egypt, Iraq, New Zealand, Sweden and Thailand, World Health Assembly Agenda Item: 8.4’, 21 May 1955, http://apps.who.int/iris/bitstream/10665/103853/1/WHA8_AFL-26_eng.pdf (consulted 20 October 2017).

94

WHO, The work of WHO: annual report of the Director-General to the World Health Assembly and to the United Nations, 1964, Geneva: WHO, 1965, p. 66, http://apps.who.int/iris/handle/10665/85775 (consulted 20 October 2017).

95 WHO, MH/D/9.54, Consultative Committee on Public Information, Twentieth session, item 5, 25 June 1954: ‘Report on WHO public information activities during the period November 1953 and June 1954’.

96 WHO, *Work of WHO: annual report 1955*, p. 53.

97 WHO, *Work of WHO: annual report 1964*, p. 66.

98
*Life International*, August 1958, pp. 68–75.

99 Fred Clarke, ‘500 million children I can’t forget’, *The Rotarian*, May 1958, pp. 49–51.

100 This was not without controversy, however, when the information had been deliberately inserted. See Alleyne, *Global lies?*, pp. 68–9.

101 WHO Archives, Photographic Archives, Sub-fonds Photographers, Cancer, Kenya, Almasy, 1972, letter to Tibor Farkas, 29 June 1973.

102 See, for instance, WHO Archives, General Aspects of Film Production, Policy, Distribution, V/6/158/1.

103

LangloisSuzanne, ‘And action! UN and UNESCO coordinating information films, 1945–1951’, in Poul Duedahl, ed., A history of UNESCO: global actions and impacts, London: Palgrave Macmillan, 2016, p. 73 ; PattersonRichard, ‘The UN in Hollywood: a lesson in public relations’, Hollywood Quarterly, 5, 4, 1951, p. 327 .

104 Patterson, ‘UN in Hollywood’, pp. 328–9.

105

ScottIan, ‘The glass wall: ideology, Cold War history and post-9/11 Hollywood reflections of the United Nations’, Journal of Popular Film and Television, 44, 2, 2016, p. 81 ; Patterson, ‘UN in Hollywood’, pp. 329–32.

106 United Nations Film Board, 16th session, summary record, 1951 – UNESDOC, p. 3, http://unesdoc.unesco.org/images/0017/001787/178798eb.pdf (consulted 20 October 2017).

107 WHO Archives, General Aspects of Film Production, Policy, Distribution, V/6/158/1.

108 WHO, *Work of WHO: annual report 1948*, pp. 37–8.

109 WHO, *Second ten years*, pp. 291–2.

110

WHO, The work of WHO: annual report of the Director-General to the World Health Assembly and to the United Nations, 1959, Geneva: WHO, 1960, p. 50, http://apps.who.int/iris/handle/10665/85722 (consulted 20 October 2017).

111

WHO, The work of WHO: annual report of the Director-General to the World Health Assembly and to the United Nations, 1952, Geneva: WHO, 1953, p. 61, http://apps.who.int/iris/handle/10665/85644 (consulted 20 October 2017).

112

HatfieldKatherine, HinckAshley and BirkholtMarty J., ‘Seeing the visual in argumentation: a rhetorical analysis of UNICEF Belgium’s Smurf public service announcement’, Argumentation and Advocacy, 43, 3–4, 2007, pp. 144–151 ; AliSadaf Rashid, JamesDebbie, and VulteeFred, ‘Strike a pose: comparing associated press and UNICEF visual representations of the children of Darfur’, African Conflict & Peacebuilding Review, 3, 1, 2013, pp. 1–26 ; Fehrenbach and Rodogno, ‘Humanitarian photography’, p. 1132.

113 WHO Archives, Co-ordination with UN and specialized agencies – World Population Year – 1974, P13/372/11, Jacket 1, CESI/PC.71-17, 27 August 1971, p. 14.

114

CapaCornell, ed., The concerned photographer 2, London: Thames & Hudson, 1972, p. 1 .

115

CuetoMarcos, ‘The origins of primary health care and selective primary health care’, American Journal of Public Health, 94, 11, 2004, pp. 1864–1874 1551422110.2105/ajph.94.11.1864PMC1448553; ChorevNitsan, The World Health Organization between north and south, Ithaca, NY: Cornell University Press, 2012, pp. 124–150 .

116 WHO, *Proposed programme budget for 1978 and 1979*, Geneva: World Health Organization, 1976, p. 318, http://apps.who.int/iris/handle/10665/86032 (consulted 20 October 2017).

